# Two Different Copy Number Variations of the *CLCN2* Gene in Chinese Cattle and Their Association with Growth Traits

**DOI:** 10.3390/ani12010041

**Published:** 2021-12-26

**Authors:** Jia Tang, Xuemei Shen, Yu Yang, Haiyan Yang, Ao Qi, Shuling Yang, Kaixing Qu, Xianyong Lan, Bizhi Huang, Hong Chen

**Affiliations:** 1 Key Laboratory of Animal Genetics, Breeding and Reproduction of Shaanxi Province, College of Animal Science and Technology, Northwest A&F University, Xianyang 712100, China; candy2038@nwafu.edu.cn (J.T.); shenxuemei@nwafu.edu.cn (X.S.); yangyu921048495@nwafu.edu.cn (Y.Y.); yanghaiyan0316@nwafu.edu.cn (H.Y.); 18734453163@nwafu.edu.cn (A.Q.); yangshuling@nwafu.edu.cn (S.Y.); lan342@126.com (X.L.); 2 Academy of Science and Technology, Chuxiong Normal University, Chuxiong 675000, China; kaixqu@163.com; 3 Yunnan Academy of Grassland and Animal Science, Kunming 650212, China; 4 College of Animal Science, Xinjiang Agricultural University, Urumqi 830052, China

**Keywords:** CNV, chloride voltage-gated channel 2, bovine, body size data

## Abstract

**Simple Summary:**

Compared with traditional breeding methods, molecular marker breeding can greatly speed up the process of livestock breeding to improve the production performance of economic traits. As one form of genetic variation, copy number variations could be used for molecular marker breeding. This study explored CNVs in bovine CLCN2 genes on account of this gene possibly being involved in cell volume regulation, transepithelial transport and cell proliferation. We investigated their association with growth traits in four Chinese cattle breeds. Our results provide evidence that there are two different copy number variations in CLCN2, which are associated with growth traits in two different Chinese cattle populations and could be used as candidate markers for cattle molecular breeding.

**Abstract:**

Copy number variation (CNV) can affect gene function and even individual phenotypic traits by changing the transcription and translation level of related genes, and it also plays an important role in species evolution. Chloride voltage-gated channel 2 (*CLCN2*) encodes a voltage-gated chloride channel (CLC-2), which has a wide organ distribution and is ubiquitously expressed. Based on previous studies, we hypothesize that *CLCN2* could be a candidate gene involved in cell volume regulation, transepithelial transport and cell proliferation. This study aimed to explore CNVs in the *CLCN2* gene and investigate its association with growth traits in four Chinese cattle breeds (Yunling cattle, Xianan cattle, Qinchuan cattle and Pinan cattle). We identified there are two copy number variation regions (CNV1: 3600 bp, including exon 2–11; CNV2: 4800 bp, including exon 21–22) of the *CLCN2* gene. The statistical analysis showed that the CNV1 mutation in the YL cattle population was significantly associated with cannon circumference (*p* < 0.01). The CNV2 mutation in the XN cattle population had a significant effect on body slanting length, chest girth and body weight (*p* < 0.05). In the YL cattle, the association analysis of *CLCN2* gene CNV1 and CNV2 combination with cannon circumference was significant (*p* < 0.01). Our results provide evidence that CNV1 and CNV2 in *CLCN2* are associated with growth traits in two different cattle populations and could be used as candidate markers for cattle molecular breeding.

## 1. Introduction

Beef is an important food source of protein for human beings. In recent years, the quantity and quality of beef consumption have been greatly improved in China [1]. Continuous breeding can improve the production performance of economic traits, while the traditional breeding methods usually need several generations and a long process. Molecular marker breeding can greatly accelerate the breeding process of livestock [2]. Various genes regulate meat traits, and we can study their genetic variations for marker assisted selection [3]. As a new form of genetic variation, copy number variation (CNV) is defined as a large-scale structural variation between 50 bp and 5 Mb in the genome, including the insertion, deletion, duplication and complex mutations at multiple chromosomal sites [4]. Compared with single nucleotide polymorphism (SNP), copy number variation has fewer variation sites and longer sequences, making it easier to detect and study. CNV can affect gene function and even individual phenotypic traits by changing the transcription and translation levels of related genes, and it also plays an important role in species evolution. The genomic CNV data of several breeds of cattle have been published [5,6], showing that CNV is closely related to the health status and economic traits of cattle. Therefore, it has broad prospects and is of great significance to speed up the process of breeding improvement.

Chloride voltage-gated channel 2 (*CLCN2*) encodes a voltage-gated chloride channel (CLC-2), which has a wide organ distribution and is ubiquitously expressed. The encoded protein is a transmembrane protein that maintains chloride ion homeostasis in various cells and is critical for electrogenesis, homeostatic control of cell volume, and maintenance of ion gradients [7]. Volume-activated chloride channels (VACCs) may participate in cell proliferation and apoptosis [8] through cell swelling as well as shrinkage [9] and is involved in migratory capacity and cell proliferation [10]. Many studies have proved that some Cl^−^ channel blockers affect the proliferation of a variety of cell types, such as endothelial cells [11], vascular smooth muscle cells [12], glioma cells [13], intestinal enterocytes [14] and human leukemic cells [15]. Meanwhile, the current study indicates that voltage-gated CLC-2 may serve as an important positive regulator in oligodendrocyte precursor cell differentiation by regulating the expression of myelin gene transcription factors [16]. Studies have provided data to demonstrate that *CLCN2* plays a role in the IGF-1-induced regulation of vascular smooth muscle cell proliferation in cardiovascular diseases [17]. It might also exert functions via the PI3K/AKT and the Wnt/β-catenin signaling pathways [18,19]. Previous studies have pointed out that the function and regulation of ClC-2 are highly conservative among different species [20]. From the above, we hypothesize that the *CLCN2* gene could be a candidate gene for involvement in cell volume regulation, transepithelial transport and cell proliferation [21].

There are several prominent beef cattle breeds in China, and each breed has a specific genetic background. Yunling cattle (YL) is the first cattle breed bred by ternary cross breeding including Brahman, Murray Grey and Yunnan native cattle. Xianan cattle (XN) is the cultivate breed hybrid of French Charolais cattle and Nanyang cattle, which was the first beef cattle breed in China. Qinchuan cattle (QC) is one of China’s five major varieties and has become an important source of increased income for farmers in many Chinese provinces. Pinan cattle (PN) is a hybrid between Piedmont cattle and Nanyang cattle in China with excellent performance that has much room for improvement. In brief, these cattle breeds play an irreplaceable role in China’s animal husbandry industry and have great potential.

In this study, we analyzed the associations between CNVs (CNV1: 3600 bp, including exon 2–11; CNV2: 4800 bp, including 21–22 exon) of the *CLCN2* gene and the growth traits of Yunling cattle, Xianan cattle, Qinchuan cattle and Pinan cattle. The results show that CNVs of *CLCN2* gene could be used as candidate markers for cattle molecular breeding.

## 2. Materials and Methods

### 2.1. Animals and Growth Traits Measurements

The study used the blood samples of adult female cattle, which were freely-fed with corn silage. We selected a total of 573 cattle from four kinds of cattle to study the copy number variations of the *CLCN2* gene: Yunling cattle (YL, *n* = 176, Kunming City, Yunnan Province, China), Xianan cattle (XN, *n* = 97, Zhumadian City, Henan Province, China), Qinchuan cattle (QC, *n* = 34, Baoji City, Shaanxi Province, China), Pinan cattle (PN, *n* = 266, Nanyang City, Henan Province, China).

We measured the corresponding growth traits of these 573 cattle. For YL, we measured body height (BH), hip height (HH), body slanting length (BSL), chest girth (CG), abdominal girth (AG), cannon circumference (CC), chest width (CW), chest depth (CD), hip girth (HG), hucklebone width (HBW), hip width (HW), rump length (RL) and body weight (BW). In the XN population, body height (BH), hip height (HH), body slanting length (BSL), chest girth (CG), abdominal girth (AG), cannon circumference (CC) and body weight (BW) were measured. For QC, we measured body height (BH), hip height (HH), body length (BL), chest girth (CG), chest width (CW), chest depth (CD), rump length (RL), hip width (HW), hucklebone width (HBW) and body weight (BW). In the PN population, body height (BH), body slanting length (BSL), hip height (HH), chest girth (CG), hucklebone width (HBW) and rump length (RL) were measured.

### 2.2. Preparation of Sample and Genomic DNA

This study collected all the blood samples of the cattle and used a standard phenol–chloroform protocol to extract genomic DNA [22]. Nanodrop 2000 was used to determine the quantity of genomic DNA. Then, the DNA samples were diluted to 25 ng/µL and stored at −40 °C.

### 2.3. Candidate Gene Identification and Primer Design

**Figure 1:**
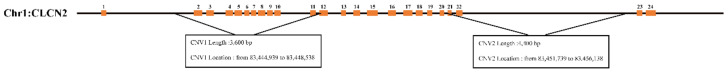
Information about the two CNV regions of *CLCN2* gene in cattle breeds.

**Figure 2:**
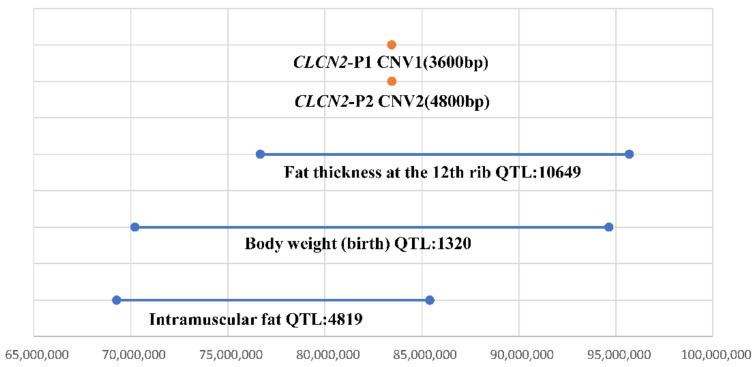
The two CNV regions of the *CLCN2* gene overlap with the QTLs of the cattle.

According to existing comparative genomic hybridization (CGH) analysis results, we identified two copy number variation regions in the *CLCN2* gene (AC_000158.1) located at Chr1:83,444,939 to 83,448,538 bp (3600 bp) and Chr1:83,451,739 to 83,456,138 bp (4400 bp) in Chinese cattle. Information about the *CLCN2* gene primers is shown in Figure 1 and Table 1. The orange boxes in Figure 1 represent coding regions. All primers were designed by NCBI primer blast. We found that the *CLCN2* gene was located in QTLs associated with intramuscular fat, body weight (birth) and fat thickness at the 12th rib (Figure 2).

### 2.4. Detection of the CLCN2 Gene Copy Number

In this study, quantitative polymerase chain reaction (qPCR) was used to determine the copy number of potential CNVs. The bovine basic transcription factor 3 (*BTF3*) gene was the housekeeping gene. The 10 µL reaction mixtures contained 25 ng of genomic DNA, 0.5 μL of primers, 5 µL of ChamQ SYBR qPCR Master Mix (Vazyme, Nanjing, China) and 3 µL of ${\mathrm{ddH}}_{2}O$. The thermal cycling conditions were 95 °C for 30 s followed by 40 cycles of 95 °C for 10 s and 60 °C for 30 s.

### 2.5. Statistical Analysis

In this study, the copy number (CN) of the *CLCN2* gene was analyzed using $2\times 2^{-\Delta \Delta Ct}$ [23,24]. The copy number was divided into three types for each region: loss (CN < 2), median (CN = 2) and gain (CN > 2). Thus, there were nine types of the copy number of the *CLCN2* CNV1 and CNV2 combination: loss and loss (CN1 < 2, CN2 < 2), loss and normal (CN1 < 2, CN2 = 2), loss and gain (CN1 < 2, CN2 > 2), normal and loss (CN1 = 2, CN2 < 2), normal and normal (CN1 = 2, CN2 = 2), normal and gain (CN1 = 2, CN2 > 2), gain and loss (CN1 > 2, CN2 < 2), gain and normal (CN1 > 2, CN2 = 2), gain and gain (CN1 > 2, CN2 > 2). We analyzed the associations of *CLCN2* CNVs with growth traits in YL, XN, QC and PN cattle using the one-way analysis of variance (ANOVA) technique in IBM SPSS Statistics 23 software. On the basis of different factors affecting growth traits in cattle, considering age and genetic effects, we used the general linear model method, and predigested for the current situation. The finished model employed Equation (1), in which $Y_{ijk}$ is the record of individual phenotypes, µ is population mean, $A_{i}$ is the effect due to age, $CNV_{j}$ is the fixed effect of CNV type of the *CLCN2* gene and $e_{\mathrm{ijk}}$ is random error.

(1) $$Y_{ijk}=\mu +A_{i}+CNV_{j}+e_{ijk}$$

## 3. Results

### 3.1. Distribution of CNVs of CLCN2 Gene in the Experimental Sample Group

**Figure 3:**
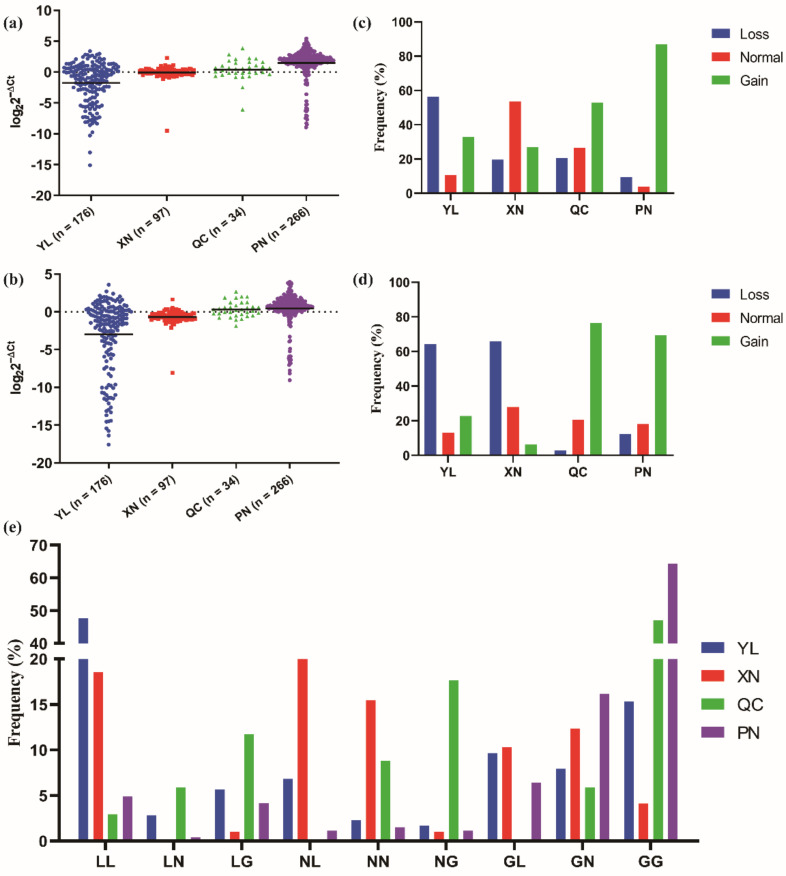
The distribution of the *CLCN2* gene CNV1 and CNV2 and their combination in the different Chinese cattle breeds. (**a**) The distribution of *CLCN2* gene CNV1 copy numbers in four Chinese cattle breeds. (**b**) The distribution of *CLCN2* gene CNV2 copy numbers in four Chinese cattle breeds. (**c**) The frequency of the copy numbers of the *CLCN2* gene CNV1 in four Chinese cattle breeds. (**d**) The frequency of the copy numbers of the *CLCN2* gene CNV2 in four Chinese cattle breeds. (**e**) The frequency of the copy numbers of the *CLCN2* gene CNV1 and CNV2 combination in four Chinese cattle breeds. LL means loss and loss; LN means loss and normal; LG means loss and gain; NL means normal and loss; NN means normal and normal; NG means normal and gain; GL means gain and loss; GN means gain and normal; GG means gain and gain. YL—Yunling cattle; XN—Xianan cattle; QC—Qinchuan cattle; PN—Pinan cattle.

To study the distribution of *CLCN2* CNVs in different Chinese cattle breeds, we detected the copy number of *CLCN2* in four cattle breeds, including YL, XN, QC, and PN. The three basic types (loss, normal and gain) and the nine combined types (LL, LN, LG, NL, NN, NG, GL, GN and GG) of CNV were classified, as mentioned above. As shown in Figure 3a,b, the results revealed the copy number of the *CLCN2* genes CNV1 and CNV2 was basically consistent in distribution. The frequency of the copy number polymorphisms of the *CLCN2* gene (Figure 3c,d) illustrated that the loss type was maximal in YL and the gain type was maximal in QC and PN; these results were similar for both CNV1 and CNV2. Therefore, as the frequency of the copy numbers of the *CLCN2* gene CNV1 and CNV2 combination shows, the loss and loss type was the main type in YL and the gain and gain type was the main type in QC and PN. For XN, the main type was the normal type in CNV1, the loss type in CNV2, and the normal and loss type in the CNV1 and CNV2 combination (Figure 3e).

### 3.2. Association Analysis

In this study, we analyzed the association of the *CLCN2* gene copy number types with the growth traits of four Chinese cattle breeds using a general linear model. The association showed that that the CNV1 mutation in the YL cattle population significantly affected cannon circumference (*p* = 0.002), as well as that cattle with the loss type of CNV1 had higher cannon circumference than those with the normal or gain type (Table 2). The CNV2 mutation in the XN cattle population had a significant effect on body slanting length (*p* = 0.031), chest girth (*p =* 0.025) and body weight (*p* = 0.034), which were higher for individuals with the normal type of CNV2 than with the loss or gain type (Table 3). In YL cattle, the association analysis of the *CLCN2* gene CNV1 and CNV2 combination with growth traits showed cattle with the loss and normal type had significantly better traits than those with other types in cannon circumference (*p* = 0.006) (Table 4).

## 4. Discussion

Genetic variation takes many forms, ranging from large chromosome anomalies to single nucleotide changes. Various studies have discovered an abundance of copy number variations of DNA segments varying from kilobases (kb) to megabases (Mb) in size [25,26]. With the latest development of high throughput sequencing technology, a larger number of CNVs have been identified in many animal husbandry species, such as chicken [27], pig [28,29], cattle [30,31] and sheep [32]. By molecular mechanisms, such as gene disruption, gene fusion, positive effect and dosage effect, CNVs could be associated with complex disease or quantitative traits [33]. Many studies have worked on the CNV-associated, economically important traits in the genomes of livestock [34,35].

The *CLCN2* gene is expressed in many different tissues and its physiological functions are quite diverse, including transepithelial fluid transport, cell volume regulation and neuronal chloride homeostasis regulation [21,36,37]. Chloride channels modify cytoplasmic Cl^−^ activity and mediate osmolyte flux, thus affecting cell volume. Most chloride channels allow the exit of HCO_3_^−^, leading to cytosolic acidification, thereby inhibiting cell proliferation and promoting cell apoptosis [38]. In addition, it has been proved the Cl^−^ channel blockers may perturb the intracellular ionic environment, resulting in G0/G1 arrest via the p21 pathway [15]. The *CLCN2* gene is closely related to cell proliferation and apoptosis, which provides a reliable theoretical basis for the necessity of this study. However, a detailed explanation of the function of the *CLCN2* gene in Chinese cattle is lacking, making it a relatively unknown area that still needs extensive study.

In this research, we found that the CNV regions of the *CLCN2* gene overlapped with the QTL regions correlated with intramuscular fat, body weight (birth) and fat thickness at the 12th rib. Therefore, we inferred that the copy number variation of the *CLCN2* gene may be related to the growth traits of cattle. We chose four Chinese cattle breeds (Yunling cattle, Xianan cattle, Qinchuan cattle and Pinan cattle) to explore CNVs in *CLCN2* gene and investigate their association with growth traits. The results showed the CNVs of the *CLCN2* gene existed in all four Chinese cattle breeds, while the distributions of *CLCN2* copy numbers varied by variety. This may be due to the diversity of background or influenced by the growing environment. Except Qinchuan cattle, all the Chinese cattle studied were crossbred cattle from different breed combinations. On the other hand, all cattle populations were maintained in Central China, except Yunling cattle, which were raised in Southwest China. These reasons may have caused the main types of the four breeds to be different.

As for the correlation analysis, the results showed the CNV1 and CNV2 in *CLCN2* were associated with growth traits including cannon circumference, body slanting length, chest girth and body weight. Yunling cattle with a decreased copy number in CNV1 and a normal copy number in CNV2 exhibited thicker cannon circumference. Xianan cattle with the normal type of CNV2 showed better phenotypes than other types. The data suggested that CNVs of the *CLCN2* gene could be used as a molecular genetic marker of cattle, allowing improvement to the growth traits of cattle and accelerating the breeding process. Currently, studies on mutations of *CLCN2* gene investigate brain white matter edema [39], secondary paroxysmal kinesigenic dyskinesia [40], idiopathic generalized epilepsy [41] and other diseases. Most mutations affect chloride channel function and trafficking by altering the protein encoded by *CLCN2*. As a member of the chloride channel family, there was a missense mutation in *CLCN7* gene, accelerating the gating of the lysosomal Cl^−^/H^−^exchanger ClC-7/Ostm1, causing osteopetrosis with gingival hamartomas in cattle [42]. Further experimental studies are required to determine the specific mechanism of action and functional differences of copy number variations between different varieties.

## 5. Conclusions

In summary, this is the first detection and validation of the two different CNVs of the *CLCN2* gene in the four Chinese cattle populations. The loss type of CNV1 and the loss and normal type of CNV1 and CNV2 combination in YL was associated with increased cannon circumference, while the normal type of CNV2 was related to body slanting length, chest girth and body weight in XN. Thus, we can conclude that the loss and normal CNV types of the *CLCN2* gene are related to better phenotypes in these Chinese cattle breeds. Our findings revealed *CLCN2* CNVs could be used as candidate markers for cattle molecular breeding.

## Figures and Tables

**Table 1:** Primers designed for the genes used in this study.

Genes	Sequences (5′-3′)	Amplification Length
*CLCN2*-CNV1	Forward primer: TTCAGCGCCTTCATCTTCCG	104 bp
	Reverse primer: GCCCCACCTCATCTGAAACAT	
*CLCN2*-CNV2	Forward primer: TGGGGAGTCTGGGGTCTAAC	120 bp
	Reverse primer: TCCTCACCAGGATAGGGCTG	
*BTF3*	Forward primer: AACCAGGAGAAACTCGCCAA	120 bp
	Reverse primer: TTCGGTGAAATGCCCTCTCG	

**Table 2:** Association analysis of *CLCN2* gene CNV1 with growth traits in YL cattle.

Breed	Growth Traits	CNV Types (Mean ± SE)			*p*-Value
		Loss (*n* = 99)	Normal (*n* = 19)	Gain (*n* = 58)	
Yunling cattle	cannon circumference (cm)	18.88 ± 0.140 ^A^	18.47 ± 0.269 ^AB^	17.74 ± 0.360 ^B^	0.002 **

A and B denote values that differ significantly at **, *p* < 0.01.

**Table 3:** Association analysis of *CLCN2* gene CNV2 with growth traits in XN cattle.

Breed	Growth Traits	CNV types (Mean ± SE)			*p*-Value
		Loss (*n* = 64)	Normal (*n* = 27)	Gain (*n* = 6)	
Xianan cattle	body slanting length (cm)	158.09 ± 0.707 ^a^	161.59 ± 1.591 ^b^	155.50 ± 2.717 ^ac^	0.031 *
	chest girth (cm)	191.61 ± 1.019 ^a^	196.67 ± 1.768 ^b^	189.83 ± 3.156 ^ab^	0.025 *
	body weight (kg)	543.19 ± 6.694 ^a^	574.48 ± 13.627 ^b^	523.00 ± 11.897 ^ab^	0.034 *

a, b and c denote values that differ significantly at *, *p* < 0.05.

**Table 4:** Association analysis of *CLCN2* gene CNV1 and CNV2 combination with growth traits in YL cattle.

Breed	Growth Traits	CNV types (Mean ± SE)									*p*-Value
		LL (84)	LN (5)	LG (10)	NL (12)	NN (4)	NG (3)	GL (17)	GN (14)	GG (27)	
Yunling cattle	cannon circumference (cm)	18.84 ± 0.147 ^Aab^	19.80 ± 0.917 ^Aab^	18.80 ± 0.442 ^Aab^	18.33 ± 0.284 ^ABa^	18.75 ± 0.946 ^ABa^	18.67 ± 0.667 ^ABab^	18.06 ± 0.369 ^ABa^	16.50 ± 1.300 ^Bb^	18.19 ± 0.283 ^ABa^	0.006 **

a and b denote values that differ significantly, *p* < 0.05; A and B denote values that differ significantly at **, *p* < 0.01. LL means loss and loss; LN means loss and normal; LG means loss and gain; NL means normal and loss; NN means normal and normal; NG means normal and gain; GL means gain and loss; GN means gain and normal; GG means gain and gain.

## Data Availability

The study did not report any data.
